# On-line Randomized Controlled Trial of an Internet Based Psychologically Enhanced Intervention for People with Hazardous Alcohol Consumption

**DOI:** 10.1371/journal.pone.0014740

**Published:** 2011-03-09

**Authors:** Paul Wallace, Elizabeth Murray, Jim McCambridge, Zarnie Khadjesari, Ian R. White, Simon G. Thompson, Eleftheria Kalaitzaki, Christine Godfrey, Stuart Linke

**Affiliations:** 1 E-health Unit, Research Department of Primary Care and Population Health, University College London, London, United Kingdom; 2 Centre for Research on Drugs and Health Behaviour, London School of Hygiene & Tropical Medicine, London, United Kingdom; 3 Medical Research Council Biostatistics Unit, Institute of Public Health, Cambridge, United Kingdom; 4 Institute of Cancer Research, Royal Cancer Hospital, London, United Kingdom; 5 Department of Health Sciences, University of York, York, United Kingdom; 6 Department of Psychology, Camden and Islington NHS Trust, London, United Kingdom; University of Cape Town, South Africa

## Abstract

**Background:**

Interventions delivered via the Internet have the potential to address the problem of hazardous alcohol consumption at minimal incremental cost, with potentially major public health implications. It was hypothesised that providing access to a psychologically enhanced website would result in greater reductions in drinking and related problems than giving access to a typical alcohol website simply providing information on potential harms of alcohol. DYD-RCT Trial registration: ISRCTN 31070347.

**Methodology/Principal Findings:**

A two-arm randomised controlled trial was conducted entirely on-line through the Down Your Drink (DYD) website. A total of 7935 individuals who screened positive for hazardous alcohol consumption were recruited and randomized. At entry to the trial, the geometric mean reported past week alcohol consumption was 46.0 (SD 31.2) units. Consumption levels reduced substantially in both groups at the principal 3 month assessment point to an average of 26.0 (SD 22.3) units. Similar changes were reported at 1 month and 12 months. There were no significant differences between the groups for either alcohol consumption at 3 months (intervention: control ratio of geometric means 1.03, 95% CI 0.97 to 1.10) or for this outcome and the main secondary outcomes at any of the assessments. The results were not materially changed following imputation of missing values, nor was there any evidence that the impact of the intervention varied with baseline measures or level of exposure to the intervention.

**Conclusions/Significance:**

Findings did not provide support for the hypothesis that access to a psychologically enhanced website confers additional benefit over standard practice and indicate the need for further research to optimise the effectiveness of Internet-based behavioural interventions. The trial demonstrates a widespread and potentially sustainable demand for Internet based interventions for people with hazardous alcohol consumption, which could be delivered internationally.

**Trial Registration:**

Controlled-Trials.com ISRCTN31070347

## Introduction

Hazardous alcohol consumption is a significant public health problem, with an estimated 3.8% of all global deaths and 4.6% of global disability-adjusted life years lost attributable to alcohol [1]. The European Union (EU) is the heaviest drinking region of the world, drinking an average of 11 litres of pure alcohol per adult each year [2]. In the UK, deaths from cirrhosis are rising, and in some age groups the increase has been nearly 10 fold over one generation of 30 years [3]. Despite the strong evidence supporting use of brief and less intensive interventions in people with alcohol use disorders, only a small minority actually receive help. Data from the Alcohol Needs Assessment Research Project indicates that in the UK fewer than 1 in 18 people with an alcohol misuse disorder access appropriate treatment, due to a combination of missed screening opportunities, limited availability of appropriate alcohol services, stigma associated with access and the wish to resolve problems alone [4]. Psychologically enhanced interventions delivered via the Internet could address all of these factors at minimal incremental cost, with potentially major public health implications.

Population access to the Internet is increasing rapidly, and in 2009 penetration was estimated to be 77% in the UK, 64% in the EU as a whole, and 74% in the US [5]. Psychologically enhanced web-based interventions make use of digital technologies to deliver a range of tailored behavioural techniques via the Internet, and have been shown to be associated with improved knowledge, self-efficacy, perceived social support, health behaviours and clinical outcomes [6]. There is growing evidence about the use of the Internet to deliver smoking cessation interventions, where automated, self-help interventions tested in on-line randomized controlled trials have recruited large numbers of participants and yielded differences in abstinence rates ranging from 8% to 20% [7]. Despite evidence that large numbers of people with risky drinking behaviours access Internet based interactive interventions [8], research in this area has been limited, with most studies employing brief normative feedback to college student samples recruited off-line [9], [10]. Additionally small trials of on-line interventions in adult populations have recruited through advertisements in newspapers, health related web-sites and telephone population surveys [11]–[13].

On-line trials can have major advantages over traditional face-to-face studies. Once the development costs have been met, they have minimal incremental running costs thus offering the ability to recruit very large numbers of participants. Different components of Internet technology allow rapid assessment, recruitment and randomisation, instantaneous collection of standardised and secure data, and delivery of on-line interventions in a controlled and uniform manner. Adoption of Internet based trial methods is increasing despite associated problems of high rates of attrition [14], [15]. Studies have indicated that on-line trials are most suitable when the intervention is safe, the medical disorder can be confirmed by remote means and outcome measures assessed using electronically transmissible technologies [16]. This paper reports the results of a large scale pragmatic on-line trial which satisfied all of these criteria.

The aim of the trial was to compare the relative effectiveness and cost-effectiveness of an on-line, psychologically enhanced, interactive computer-based intervention (DownYourDrink, DYD) in reducing alcohol consumption with a flat, text-based information website in hazardous and harmful drinkers. The objectives were to:

- Determine the effectiveness of DYD in enabling users to reduce their total alcohol consumption;
- Determine the effectiveness of DYD in reducing alcohol related harm in users;
- Determine the costs associated with the development and use of DYD;
- Determine the cost-effectiveness of DYD as a public health intervention.

## Methods

The protocol for this trial and supporting CONSORT checklist are available as supporting information; see Checklist S1 and Protocol S1.

### Design

**Figure 1:**
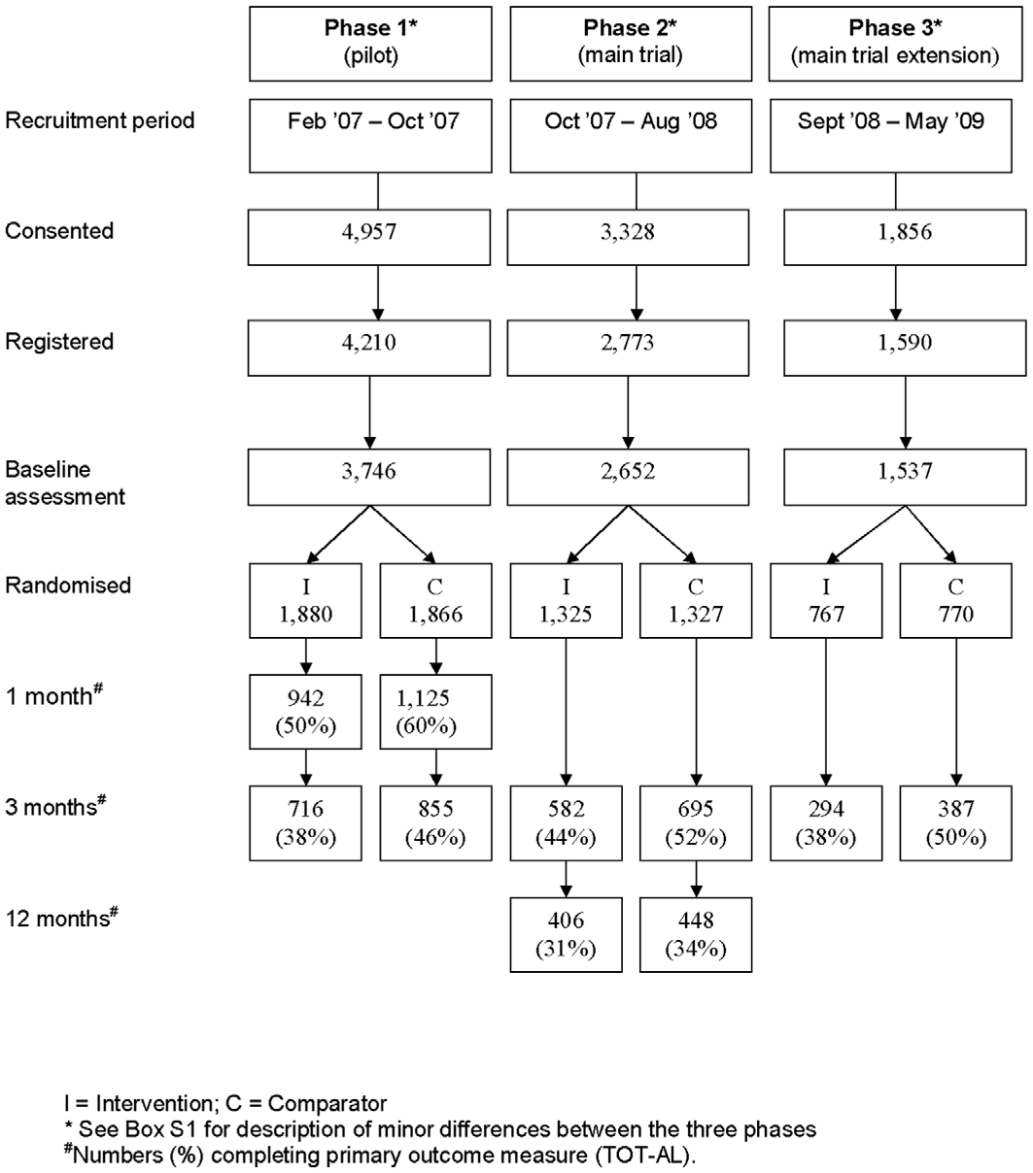
CONSORT diagram.

A two-arm individually randomised controlled trial for people with hazardous alcohol consumption was undertaken entirely on-line [17](Protocol S1). It was conducted in three phases: pilot, main trial and main trial extension (Figure 1). There were only minor differences in design between each phase (Box S1), and as these were deemed unlikely to affect outcomes materially, analysis was undertaken on data pooled from all three phases.

### Ethics and data protection

This study was conducted according to the principles expressed in the Declaration of Helsinki. Ethics approval for the study was granted by the University College London Research Ethics Committee, and all data were kept in accordance with provision of the UK Data Protection Act 1998. All patients provided written informed consent for the collection of data and subsequent analysis.

### Trial registration number

SRCTN 31070347

### Intervention and comparator websites

For the duration of the trial, both the intervention and the comparator websites were located at a single website address: www.downyourdrink.org.uk. The intervention website, hereafter known as DownYourDrink, or DYD, was a theoretically informed programme, based on brief intervention and psychological treatment principles. It offered three phases, each of which was divided into levels with different materials and associated exercises and tasks. If followed in order they provided a natural progression through three stages: decision making (Phase 1, *“It's up to you”*); implementing change (Phase 2, *“Making the change”*); and relapse prevention (Phase 3, *“Keeping on track”*). However, users were free to design their own route through the programme, and could use it as often or as seldom as they wished. Phase 1 was based on the principles of motivational enhancement therapy, phase 2 used computerised cognitive behavioural therapy and behavioural self control principles, and phase 3 was based on principles of relapse prevention. There were a number of interactive “e-tools” including a “thinking drinking diary” in which users could record their alcohol consumption along with emotional and behavioural triggers and responses. Further details about the development and content of the intervention are available elsewhere [18].

The comparator website used a similar graphical design and style to present simple, text-based information about the harms caused by excess alcohol consumption. It did not contain any interactive components, and users did not have access to the e-tools. For the duration of the trial, this comparator website was also referred to as DownYourDrink so that participants were not aware whether they had access to the intervention or comparator site.

### Recruitment

Participants were people who came across DownYourDrink while browsing the web. An earlier, simplified form of DYD had initially been launched in 2000 [19] and by the start of the trial had accrued a large number of users [8]. Most new users came to DYD from a web-search engine, such as Google or Yahoo, or from the home page of Alcohol Concern, the UK's largest alcohol charity. When users reached the home page they were invited to take a screening test (the three item Alcohol Use Disorders Identification Test or AUDIT-C [20]). Users who scored 5 or more on the AUDIT-C were informed they were potentially at risk from their alcohol consumption, and invited to join the trial. They were informed that the trial was comparing different areas of the DownYourDrink website to see which was the most effective, and that for the duration of the trial, access to DYD was only available to trial participants. Eligible participants who consented to participate were asked to register, which included providing a user name, password and valid e-mail address. This e-mail address was used to send an automated link which gave participants access to the intervention or comparator site according to their randomised allocation. The AUDIT-C scores from users who did not consent to participation in the trial were discarded automatically for ethical reasons.

Eligibility criteria were deliberately kept broad. Eligible participants were adults (aged 18 or over), scoring 5 or more on the AUDIT-C, who provided informed consent. Participants were required to have internet access. Participants who declared themselves unable to understand written English, or unwilling to complete follow-up questionnaires were excluded. People who were excluded from the trial, or who chose not to participate, were directed toward other on-line alcohol websites.

### Randomisation

Randomisation occurred in two stages. The first randomisation occurred after completion of consent and core baseline data. At this point, participants were stratified by age and gender and randomised to one of four secondary outcome measures (see below). Once all baseline measures were completed, participants were randomised to either the intervention or the comparator website. This second randomisation marked the trial entry point. Both randomisation procedures were automated, using centrally-allocated computer-generated random numbers. Thus there was no possibility of any of the trial team influencing the allocation of participants and concealment of allocation was complete.

### Outcome measures

Reactivity to assessment, or the effect of measurement itself on alcohol consumption is a well-documented phenomenon in alcohol research [21], [22]. For this reason, the total burden of assessment was kept to the minimum. All participants completed the primary outcome measure which was the TOT-AL [23]. The TOT-AL is a validated on-line measure which provided a drop-down menu for the selection of type, brand and size of beverage, and calculated the cumulative unit content of the drinks consumed over the previous 7 days (1 unit is equivalent to approximately 8 g ethanol). All participants also completed the 5 item quality of life measure, the EQ-5D [24] for the purposes of health economic analysis. We designed two single item measures to determine self-efficacy (confidence in one's ability to change behaviour) and intention, both important predictors of behaviour and intermediate variables along the pathway of change [25]. In addition, participants were asked to provide some basic demographic data at baseline (age, highest level of education attained, marital status, children, ethnicity and country of residence).

Participants were randomly allocated to one of four secondary outcome measures, each of which addressed different domains of alcohol-related harm: the Alcohol Use Disorders Test (AUDIT) [26], the Leeds Dependence Questionnaire (LDQ) [27], the Alcohol Problem Questionnaire (APQ) [28], and the ten item Clinical Outcomes in Routine Evaluation (CORE) (a measure of mental health) [29].

### Data collection

All data were collected on-line. At follow-up participants were sent an automated e-mail with an embedded hyperlink to the assessment questionnaires. Data collected at follow-up consisted of the primary outcome measure, the EQ-5D, single item measures of self-efficacy and intention, and the same secondary outcome measure completed at baseline. Up to three reminders were sent at 7 day intervals to non-responders, with the final reminder containing a request for participants to tell us their past week alcohol consumption only.

The duration of follow-up varied in the three phases of the trial. During Phase 1 (pilot), follow up was at 1 and 3 months; in the main trial follow-up was at 3 and 12 months, and in the main trial extension, follow-up was at 3 months only (Box S1). The main reasons for extending the main trial were ethical concerns. The steady recruitment, combined with unsolicited free text emails from participants, suggested that DYD was meeting a need not met by alternative services. For this reason, we were reluctant to follow our original plan which had been to make DYD unavailable to new users once our target sample size had been achieved. Equally, we could not make the intervention freely available to new users for fear of contaminating the existing trial. Hence we decided to extend recruitment to the trial, but alter the consent and follow-up procedures so that follow-up was only requested at three months. After the end of Phase 3 (main trial extension), we made the control site freely available to new users for three months, and after all data collection had been completed, made the intervention site freely available to all users.

### Statistical methods

#### Sample size calculation

A 20% reduction in past week alcohol consumption, irrespective of initial level, is typical of non-internet brief interventions [30]. In an earlier cohort study of DYD the observed mean reduction in alcohol consumption was 35% in men and 17% in women [8]. In this study the standard deviation of weekly alcohol consumption was slightly less than the mean in both men and women at both baseline and follow-up. Making a conservative assumption that the standard deviation would be equal to mean, led to the calculation that 430 participants providing follow-up data at the principal end-point in each arm would be required to give 90% power at the 5% significance level to detect a 20% difference in the past week's reported alcohol consumption between intervention and control groups [17].

#### Statistical analyses

Statistical analysis was carried out according to a pre-specified plan, comparing groups as randomised at each follow-up point. TOT-AL data were skewed and were therefore log-transformed (after adding 1 unit/week) before analysis. Means of the log-transformed data were transformed back to the original scale and are described as geometric means [31]. For those unused to geometric means, the value of the geometric mean is very similar to the value of the median. To enable comparison of our data with other alcohol intervention trials we also report the arithmetic mean in the text, as this measure has often been used in reporting trial data despite the presence of skew in the data [10]. Adjusted analyses were performed using linear regression models of outcome on randomised group, adjusting for baseline values of the respective outcome measure, AUDIT-C, age, education, self-efficacy, log (TOT-AL+1), EQ5D and gender. Missing data were handled in three stages. First, primary analyses used all available results but without imputation of missing data. Second, alternative analyses used last observation carried forward (LOCF) and multiple imputation of missing outcomes from other outcomes and website use data. Third, sensitivity analyses for missing data assumed plausible arm-specific differences between responders and non-responders [32]. Because the above analyses estimated only the effect of allocation to the intervention website, we additionally undertook a complier-average causal effect analysis to estimate the effect of compliance with the intervention [33]. This was initially performed defining compliance as more than 1 session or access to more than 10 pages within the first 3 months from randomisation, and subsequently assuming benefit to be proportional to number of page downloads and estimating the benefit of downloading 100 pages using instrumental variable methods [34]. Both these analyses used multiple imputation to handle missing outcome data.

### Health economics

Costs of the intervention included resources required in the original development of the DYD internet site and revisions undertaken for the trial by a development group comprising academics, clinicians and lay members and programmed by web consultants. Development of the control website was assumed to take a minimal proportion (5%) of overall costs. Care was taken to separate development of the intervention from research costs. Invoices for programming costs were separated into research, intervention and control costs, with 20% of the development group's time assumed to be concerned with research issues. All figures are at 2008 price levels. The primary outcome for economic evaluation was quality-adjusted life-years (QALYs) based on EQ-5D questionnaire responses valued by the UK Social Tariff valuations [35].

## Results

### Recruitment and follow-up

The recruitment period was from February 2007 until May 2009 (Figure 1). Recruitment rates were maintained throughout, averaging around 65 participants per week (Figure S1). Of the 10,141 visitors consenting to take part in the trial, 7,935 (78%) completed baseline data collection and randomisation procedures to enter the trial. At 3 months, 1,592 (40%) of the intervention group completed the TOT-AL compared with 1,937 (49%) of controls (P<0.001). Differential response rates were present across at all assessment points (Figure 1).

### Baseline assessment

**Figure 2:**
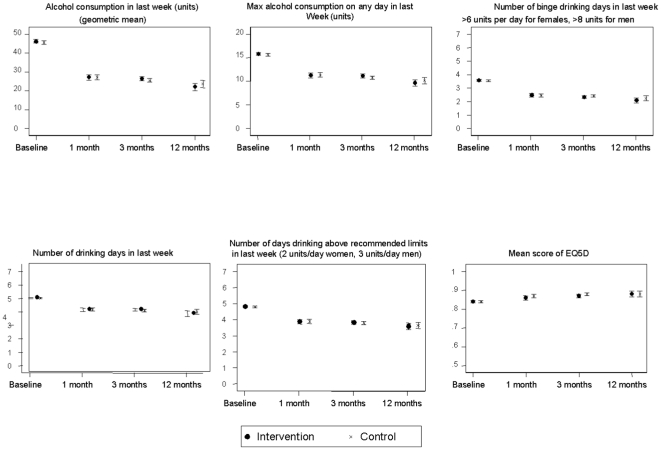
Quantity and patterns of alcohol consumption and EQ5D scores by randomized group over time: means and 95% CIs.

Although the majority of participants were White British (84%) and resident in the UK (88%), there were some from ethnic minorities, and 73 countries were represented amongst respondents. Mean age was 38 years, 57% were women and 52% were educated to at least degree level. The participants were heavy drinkers (geometric mean past week's alcohol consumption at baseline 46.0 (SD 31.2) units), drinking most days, binge drinking, and regularly drinking above recommended limits (Figure 2, baseline), but reported little evidence of dependence. There were no differences between randomized groups for any baseline characteristic (Table S1). Arithmetic mean consumption at baseline was 49.1 units for women and 68.2 units for men.

### Website usage

Participants in the intervention group made an average of 2.33 (SD 3.63) visits to the site and downloaded an average of 67 (SD 79) pages in the first month following recruitment. For the control group, the averages were 1.24 (SD 0.75) visits and 13 (SD 12) pages downloaded (p<0.001 for both visit and page comparisons) (Table S2).

### Primary outcomes

At 3 months, there was a substantial reduction in mean reported alcohol consumption in the intervention group (46.3 to 26.4 units) and the controls (45.7 units to 25.6 units). The adjusted ratio of geometric means between the two groups at 3 months was 1.03 (CI 95% 0.97 to 1.10), providing no evidence of difference between groups. Similarly, no differences were shown at 1 month or 12 months, the confidence intervals effectively ruling out the possibility of a relative reduction in mean alcohol consumption of 15% or more (Figure 2, Table 1). Similar reductions were seen in both groups at all assessment points in numbers of drinking days, days drinking above recommended limits and binge drinking occasions (Figure 2, Table S3). Arithmetic mean past week alcohol consumption for women at one, three and 12 month follow-up was 33.5, 33.1 and 27.9 units respectively, and for men intake was 48.6, 46.3 and 44.7 units at one, three and 12 months. Self-efficacy scores were higher for both groups at all follow-up assessments than at baseline. At 1 month, they were significantly higher in the intervention group than in controls, but this difference was small and not maintained at subsequent assessments. Intentions showed a slight decrease in both groups at all follow-up assessments. EQ5D scores showed little change in both groups at all assessment points (Figure 2, Table S4).

**Table 1:** Reported alcohol consumption in last week (units)# by randomised group.

	Geometric mean (SD)*		Adjusted ratio (intervention: control) of geometric means (95%CI)$
Time point**	Intervention	Control	
Baseline (n = 7,935)	46.3 (31.8)	45.7 (30.6)	-
1 month (n = 2,067)	27.1 (23.1)	27.1 (22.5)	0.98 (0.90 to 1.07)
3 months (n = 3,529)	26.4 (23.0)	25.6 (21.5)	1.03 (0.97 to 1.10)
12 months (n = 854)	22.0 (20.0)	23.5 (21.0)	0.99 (0.85 to 1.15)

# 1 unit = 8g of ethanol.

*Approximate SD back-calculated from the log scale.

**See Figure 1 for the data contributing to each time point.

$ Adjusted for baseline alcohol consumption, AUDIT-C, age, sex, education, self efficacy and EQ5D.

### Secondary outcome measures

All measures showed improvements at all follow-up assessment points for participants in both the intervention and control groups but, with the exception of LDQ at 3 months, there were no significant differences between the groups for any measure (Table S5).

### Subgroup analyses

Analyses to determine impact of pre-specified baseline characteristics (sex, educational level, baseline consumption) on past week's alcohol consumption, showed no evidence of differential effects of the intervention (all interaction P values>0.10, Table S6).

### Sensitivity analyses for missing data

Results were little changed when missing data were handled using LOCF or multiple imputation (Table S7). Sensitivity analyses allowing for systematic differences between non-responders and responders indicated that equal differences in both arms of the trial would result in little change in results, but that asymmetrical differences could produce substantial changes (Table S8).

### Effect of website exposure

In those complying with the intervention, the estimated average causal effect of allocation to intervention, expressed as a ratio of geometric means of past week's alcohol consumption, was 1.05 (95% CI 0.95 to 1.16) at 3 months. In those who downloaded 100 pages, the corresponding ratio was 1.06 (95% CI 0.94 to 1.19) (Table S9).

### Health economic analyses

The total cost of development and delivery of the DYD intervention was £107,317 and the control site cost was £3,390. These costs are detailed in Table S10. With the exception of the web maintenance costs (a small proportion of the total), these costs do not differ according to numbers accessing the site; hence the incremental costs per participant are small. The average cost per participant in the trial is £27.02 for the intervention and 85p for the control, a difference of £26.17. No significant differences in EQ5D scores or variances were found and therefore no cost-effectiveness ratio was calculated.

## Discussion

The psychologically enhanced, interactive computer-based intervention was not more effective in reducing alcohol consumption or related harms than a flat, text-based information website among hazardous and harmful drinkers. There were no differences in levels or patterns of alcohol consumption or secondary outcome measures between participants allocated to the intervention or control groups, at either the primary or secondary follow-up points. Participants in the intervention group made more use of the intervention than those in the control group, but we have no data on the relative satisfaction of the users in the two groups.

Both groups showed evidence at all follow-up points of striking improvements from baseline values in levels and patterns of alcohol consumption and in all secondary outcome measures. There are various potential explanations for these findings. Although there is clearly no difference between the effectiveness of the two interventions, it is not clear whether both interventions were effective or both were ineffective. The improvements demonstrated by trial participants could be partly due to regression to the mean (where people are motivated to join a trial at the time that their problem is most severe and through the natural history of a waxing and waning condition show an improvement over time) or to the effects of the trial assessment procedures. The therapeutic effect of assessment on alcohol consumption in trials has been well documented [21] and even minimal assessment, such as completing the 10 item AUDIT has been shown to have an effect size of 0.23 (95% CI 0.01–0.45) at 2–3 months follow-up [22]. Although we went to considerable lengths to reduce the burden of assessment it is still probable that completion of the primary outcome measure along with other aspects of study participation contributed to the observed reduction in alcohol consumption. The findings could also have been due in part to non-response bias, though this is not supported by the results of statistical analyses undertaken to deal with this anticipated aspect of the on-line trial performance. There was a marked differential in response rates between the intervention and control groups at 1 and 3 months, which had reduced but not vanished by 12 months. This differential response, with participants in the control group being more likely to respond than those in the intervention group has been seen in previous alcohol trials [36]. Our data cannot illuminate the reason for this differential, but it is possible that participants in the control group particularly welcomed the opportunity to undergo assessment, recognising this as an opportunity to reflect on their drinking behaviours.

The annual maintenance costs of DYD intervention were estimated at £12,065. Even modest recruitment rates of 50 new entrants per week evidenced in the latter stages of the trial would yield a cost of only £4.64 per person. A mean improvement in health in terms of QALYs of only 0.01 over a 12 month period would make the intervention highly cost-effective (incremental cost-effectiveness ratio of £464 per QALY). As reduced drinking is also associated with a reduction in public sector spending and improved health, such interventions taken up by those not currently accessing services could well be cost neutral and potentially significantly cost saving.

To our knowledge this is the largest pragmatic trial of an alcohol Internet intervention undertaken in the general population. It succeeded in attracting website visitors with hazardous alcohol consumption, recruiting numbers which substantially exceeded initial expectations. The study employed an innovative on-line methodology well suited to the nature of the Internet based intervention and control websites. This presented significant methodological challenges in relation both to the exclusive use of on-line assessment and to compliance with the intervention and follow-up. An extensive evidence base indicates that self reporting of alcohol consumption is at least as reliable as face to face, though uncertainty remains about the performance of these measures in on line trials [37], [38]. Many on-line trials have experienced high rates of attrition from follow-up [39] so we tested several methods to optimise response and employed a range of relevant statistical methods both to impute missing values and to estimate the effects of different levels of compliance with the intervention. Nonetheless, uncertainties remain, including the possibility of bias, as a result of the high rates of attrition from follow-up, and these need to be fully recognised in interpreting the findings.

Our results differ from previous trials of online alcohol interventions and this may reflect differences in study populations, trial procedures and comparator interventions [11]–[13]. The trial population in the present highly naturalistic study were web-browsers, whereas other studies used at least some off-line recruitment procedures, either for obtaining consent [11], or for initial identification of potential participants [12], [13]. This is likely to have implications for the study population. In this trial we used a non-interactive website which provided information about the harms of excessive alcohol consumption and advice on how to cut down. This contrasts with the Riper trial, where a pdf version of a psycho-educational brochure was used as a comparator [11]. Our decision was made partly on ethical grounds so that all participants would receive something at least as good as widely available self-help sites, and partly on research grounds to ensure trial participants were not made aware of which arm they had been randomised to.

The trial has indicated a potentially widespread and sustainable demand for Internet based interventions for people with hazardous alcohol consumption. Our findings do not provide any support for the hypothesis that psychologically enhanced interactivity confers additional benefit. However, the substantial improvement in quantity and patterns of alcohol consumption reported by participants in both arms of the trial suggests potential benefit from access to either website type, providing support for continued development and implementation of Internet applications of this kind.

## Supporting Information

Checklist S1 CONSORT Checklist.
(0.23 MB DOC)

Protocol S1 Trial Protocol.
(0.34 MB PDF)

Box S1 Differences in design between phases of the trial.
(0.04 MB DOC)

Figure S1 Cumulative recruitment to trial.
(0.01 MB TIF)

Table S1 Baseline data by randomised group.
(0.04 MB DOC)

Table S2 Use of intervention and comparator websites.
(0.03 MB DOC)

Table S3 Patterns of reported alcohol consumption over time by randomised group.
(0.04 MB DOC)

Table S4 Self-efficacy, intention and EQ5D scores over time.
(0.04 MB DOC)

Table S5 Secondary outcome measures (assessed in 1:4 participants).
(0.05 MB DOC)

Table S6 Subgroup analyses adjusting for baseline values.
(0.07 MB DOC)

Table S7 Reported alcohol consumption in last week (units) by randomised group: alternative analyses allowing for missing outcome data.
(0.04 MB DOC)

Table S8 Effect of intervention on reported alcohol consumption in last week (units): sensitivity analyses allowing for missing data.
(0.05 MB DOC)

Table S9 Causal effects of using the intervention website on reported alcohol consumption in last week (units).
(0.03 MB DOC)

Table S10 Summary of costs incurred in developing the intervention and comparator (2008 costs).
(0.03 MB DOC)
