# Supplementary material for: On-line Randomized Controlled Trial of an Internet Based Psychologically Enhanced Intervention for People with Hazardous Alcohol Consumption
Source: PLoS One. 2011 Mar 9;6(3):e14740. doi: 10.1371/journal.pone.0014740 (PMC3052303; doi:10.1371/journal.pone.0014740)
Supplement: Box S1 — Differences in design between phases of the trial. (0.04 MB DOC) [file pone.0014740.s003.doc]

| **Trial Parameter** | **Phase 1 (Pilot)** | **Phase 2 (Main trial)** | **Phase 3 (Main trial extension)** |
| --- | --- | --- | --- |
| Design | 2 arm individually randomised controlled trial | 2 arm individually randomised controlled trial | 2 arm individually randomised controlled trial |
| Intervention | Psychologically enhanced interactive website, with three phases (It’s up to you; Making the change; Keeping on Track). | Psychologically enhanced interactive website, with three phases (It’s up to you; Making the change; Keeping on Track). | Psychologically enhanced interactive website, with three phases (It’s up to you; Making the change; Keeping on Track). |
| Comparator | Text-based website with information on the harms of excess alcohol consumption | Text-based website with information on the harms of excess alcohol consumption | Text-based website with information on the harms of excess alcohol consumption |
| Recruitment | Web browsers who found the DYD home page | Web browsers who found the DYD home page | Web browsers who found the DYD home page |
| **Inclusion criteria** | Aged 18 or over;  Provided informed consent;  **Any score on AUDIT-C screen** | Aged 18 or over;  Provided informed consent;  **Score on AUDIT-C screen = 5 or more** | Aged 18 or over;  Provided informed consent;  **Score on AUDIT-C screen = 5 or more** |
| Exclusion criteria | Self-declared inability to understand written English;  Unwillingness to complete follow-up questionnaires | Self-declared inability to understand written English;  Unwillingness to complete follow-up questionnaires | Self-declared inability to understand written English;  Unwillingness to complete follow-up questionnaires |
| Randomisation | Two-stage automated randomisation, using centrally-allocated computer-generated random numbers. | Two-stage automated randomisation, using centrally-allocated computer-generated random numbers. | Two-stage automated randomisation, using centrally-allocated computer-generated random numbers. |
| **Request for off-line address and phone numbers** | **Yes** | **No** | **No** |
| **Outcome measures** | TOT-AL; EQ-5D; AUDIT; LDQ; APQ; **CORE-OM (34 items)** | TOT-AL; EQ-5D; AUDIT; LDQ; APQ; **CORE-10 (10 items)*** | TOT-AL; EQ-5D; AUDIT; LDQ; APQ; **CORE-10 (10 items)** |
| Data collection | On-line, requested by e-mail with embedded hotlink to data collection instruments. | On-line, requested by e-mail with embedded hotlink to data collection instruments. | On-line, requested by e-mail with embedded hotlink to data collection instruments. |
| **Follow-up** | **1 month and 3 months** | **3 months and 12 months** | **3 months only** |
| **Incentives** | **Not offered** | **Offered at 12 months** | **Not offered** |

Parameters with any difference between phases are shown in **bold.**

* The CORE-10 comprises 10 items drawn from the original 34 item CORE-OM. In a clinical sample (primary care) the two versions correlated at r = 0.94 (CI 0.93 to 0.95) and in a non-clinical sample (general population), the two scores correlated at r = 0.92 (CI 0.91 to 0.93) Connell, J. & Barkham, M. (2007). CORE-10 User Manual, Version 1.1. CORE System Trust & CORE Information Management Systems Ltd.
