# Supplementary material for: On-line Randomized Controlled Trial of an Internet Based Psychologically Enhanced Intervention for People with Hazardous Alcohol Consumption
Source: PLoS One. 2011 Mar 9;6(3):e14740. doi: 10.1371/journal.pone.0014740 (PMC3052303; doi:10.1371/journal.pone.0014740)
Supplement: Table S1 — Baseline data by randomised group. (0.04 MB DOC) [file pone.0014740.s005.doc]

| **Demographic variables** | **Intervention**  **N=3,972** | **Control**  **N= 3,963** |
| --- | --- | --- |
| Age (years): mean (SD) | 38 (11) | 38 (11) |
| Females: n (%) | 2,246 (57) | 2,299 (58) |
| Educated at least to degree level: n (%) | 2,069 (52) | 2,026 (51) |
| Married/long term relationship: n (%) | 2,424 (61) | 2,476 (62) |
| With children: n (%) | 2,053 (52) | 2,027 (51) |
| White British: n (%) | 3,318 (84) | 3,317 (84) |
| Living in UK: n (%) | 3,496 (88) | 3,492 (88) |
| Provided terrestrial address: n (%) | 1,408 (35) | 1,379 (35) |
| Provided phone number: n (%) | 1,232 (31) | 1,187 (30) |
| AUDIT-C: mean (SD) | 8.51 (2.02) | 8.49 (2.02) |
| Past week’s alcohol consumption (TOT-AL)  (Geometric mean and approx. SD*) | 46.3 (31.8) | 45.7 (30.6) |
| Maximum units consumed in any one day  (Geometric mean and approx. SD*) | 15.8 (9.5) | 15.6 (9.5) |
| Number of drinking days: mean (SD) | 5.0 (1.9) | 5.0 (1.9) |
| Number of drinking days drinking above recommended limits (> 2 ♀ / >3 ♂ units of alcohol): mean (SD) | 4.8 (1.9) | 4.8 (1.9) |
| Number of days binge drinking (>6 ♀ / >8 ♂ units of alcohol): mean (SD) | 3.6 (2.2) | 3.5 (2.1) |
| EQ5D: mean (SD) | 0.84 (0.19) | 0.84 (0.19) |
| Health state meter: mean (SD) | 66.6 (23.6) | 66.5 (23.4) |
| Self-efficacy score: median (IQR)  0: low, 5: high | 3 (2) | 3 (2) |
| Intentions score: median (IQR)  0: low, 5: high | 4 (2) | 4 (2) |
| AUDIT**: mean (SD) | 18.8 (7.4) | 18.7 (7.2) |
| APQ**: mean (SD) | 6.6 (4.3) | 6.7 (4.2) |
| LDQ**: mean (SD) | 9.1 (5.8) | 8.7 (5.5) |
| CORE-OM**: mean (SD) (Phase 1) | 1.3 (0.7) | 1.3 (0.7) |
| CORE-10**: mean (SD) (Phases 2 & 3) | 16.3 (4.9) | 16.6 (5.0) |

* Approximate SD back-calculated from the log scale

** Available in a 1-in-4 randomised sample.

AUDIT : Alcohol Use Disorders Test

APQ: Alcohol Problems Questionnaire,

LDQ: Leeds Dependence Questionnaire

CORE-OM and CORE-10: Measure of mental health.
