# Supplementary material for: On-line Randomized Controlled Trial of an Internet Based Psychologically Enhanced Intervention for People with Hazardous Alcohol Consumption
Source: PLoS One. 2011 Mar 9;6(3):e14740. doi: 10.1371/journal.pone.0014740 (PMC3052303; doi:10.1371/journal.pone.0014740)
Supplement: Table S2 — Use of intervention and comparator websites. (0.03 MB DOC) [file pone.0014740.s006.doc]

|  | **Phase 1**  **N=3,746** | **Phase 2**  **N=2,652** | **Phase 3**  **N=1,537** |
| --- | --- | --- | --- |
| **Website Sessions** |  |  |  |
| Overall | 1.77 (2.45) | 1.76 (2.86) | 1.88 (2.88) |
| Intervention | 2.24 (3.28) | 2.32 (3.90) | 2.57 (3.91) |
| Control | 1.29 (0.84) | 1.19 (0.69) | 1.18 (0.62) |
| **Pages downloaded** |  |  |  |
| Overall | 38 (55) | 39 (54) | 42 (60) |
| Intervention | 63 (68) | 64 (67) | 73 (73) |
| Control | 13 (13) | 13 (12) | 12 (11) |

Number of website sessions and pages downloaded at 3 months by randomised group
