# Supplementary material for: On-line Randomized Controlled Trial of an Internet Based Psychologically Enhanced Intervention for People with Hazardous Alcohol Consumption
Source: PLoS One. 2011 Mar 9;6(3):e14740. doi: 10.1371/journal.pone.0014740 (PMC3052303; doi:10.1371/journal.pone.0014740)
Supplement: Table S3 — Patterns of reported alcohol consumption over time by randomised group. (0.04 MB DOC) [file pone.0014740.s007.doc]

| **Patterns of alcohol consumption** | **Time point** | **Mean (SD)** | | **Adjusted difference (intervention-control) of means (95%CI)^$^** |
| --- | --- | --- | --- | --- |
|  |  | **Intervention** | **Control** |  |
| **Number of drinking days** | Baseline n=7,935 | 5.0 (2) | 5.0 (2) | - |
|  | 1 month n=2,067 | 4.2 (2) | 4.2 (2) | -0.13 (-0.28 to 0.02) |
|  | 3 months n=3,529 | 4.1 (2) | 4.1 (2) | 0.02 (-0.10 to 0.15) |
|  | 12 months n=854 | 3.9 (2) | 4.0 (2) | -0.07 (-0.32 to 0.19) |
| **Number of days above recommended limits (>2 ♀ / > 3+ ♂ units of alcohol a day)** | Baseline n=7,935 | 4.8 (2) | 4.8 (2) | - |
|  | 1 month n=2,067 | 3.9 (2) | 3.9 (2) | -0.10 (0.25 to 0.06) |
|  | 3 months n=3,529 | 3.8 (2) | 3.8 (2) | 0.01 (-0.12 to 0.13) |
|  | 12 months n=854 | 3.6 (2) | 3.7 (2) | 0.03 (-0.23 to 0.29) |
| **Number of days binge drinking (>6 ♀ / 8+ ♂ units of alcohol)** | Baseline n=7,935 | 3.6 (2) | 3.5 (2) | - |
|  | 1 month n=2,067 | 2.5 (2) | 2.5 (2) | -0.03 (-0.18 to 0.12) |
|  | 3 months n=3,529 | 2.3 (2) | 2.4 (2) | -0.07 (-0.18 to 0.05) |
|  | 12 months n=854 | 2.1 (2) | 2.2 (2) | 0.00 (-0.22 to 0.22) |
|  |  | Geometric mean (SD) | | **Adjusted ratio (intervention / control) of geometric means (95%CI)^$^** |
| **Max units consumed on any 1 day** | Baseline n=7,935 | 15.8 (9) | 15.7 (10) | - |
|  | 1 month n=2,067 | 11.3 (9) | 11.4 (8) | 0.99 (0.93 to 1.05) |
|  | 3 months n=3,529 | 11.1 (7) | 10.8 (9) | 1.04 (0.99 to 1.09) |
|  | 12 months n=854 | 9.7 (7) | 10.1 (7) | 0.99 (0.89 to 1.10) |

^$^ Adjusted for baseline alcohol consumption, AUDIT-C, age, sex, education, self-efficacy and EQ5D
