# Supplementary material for: On-line Randomized Controlled Trial of an Internet Based Psychologically Enhanced Intervention for People with Hazardous Alcohol Consumption
Source: PLoS One. 2011 Mar 9;6(3):e14740. doi: 10.1371/journal.pone.0014740 (PMC3052303; doi:10.1371/journal.pone.0014740)
Supplement: Table S4 — Self-efficacy, intention and EQ5D scores over time. (0.04 MB DOC) [file pone.0014740.s008.doc]

| **Outcome measure** | **Time point** | **Mean (SD)** | | **Adjusted difference (intervention-control) of means (95%CI)^$^** |
| --- | --- | --- | --- | --- |
|  |  | **Intervention** | **Control** |  |
| **Self –efficacy**  **(0 = low, 5 = high)** | Baseline n=7,935 | 2.77 (1.16) | 2.79 (1.15) | - |
|  | 1 month n=2,001 | 3.31 (1.17) | 3.16 (1.18) | **0.16 (0.08 to 0.25)** |
|  | 3 months n=3,434 | 3.28 (1.21) | 3.25 (1.20) | 0.02 (-0.05 to 0.09) |
|  | 12 months n=841 | 3.41 (1.18) | 3.33 (1.23) | 0.05 (-0.10 to 0.20) |
| **Intentions**  **(0 = low, 5 = high)** | Baseline n=7,935 | 3.80 (1.11) | 3.79 (1.10) | - |
|  | 1 month n=2,001 | 3.68 (1.15) | 3.62 (1.15) | 0.05 (-0.03 to 0.13) |
|  | 3 months n=3,434 | 3.65 (1.18) | 3.61 (1.17) | 0.06 (-0.01 to 0.13) |
|  | 12 months n=841 | 3.61 (1.18) | 3.60 (1.17) | 0.03 (-0.11 to 0.17) |
| **Health state meter (0 = dead, 100 = best possible)** | Baseline n=7,935 | 66.6 (24) | 66.5 (23) | - |
|  | 1 month n=2,117 | 72.2 (20) | 72.7 (20) | -0.29 (-1.77 to 1.16) |
|  | 3 months n=3,621 | 71.4 (22) | 72.1 (21) | -0.85 (-2.09 to 0.41) |
|  | 12 months n=867 | 72.4 (21) | 72.4 (21) | -0.61 (-3.11 to 1.88) |
| **EQ5D score**  **(0 = low, 1 = high)** | Baseline n=7,935 | 0.84 (0.19) | 0.84 (0.19) | - |
|  | 1 month n=2,122 | 0.86 (0.18) | 0.87 (0.17) | -0.01 (-0.02 to 0.00) |
|  | 3 months n=3,629 | 0.87 (0.18) | 0.88 (0.17) | -0.01 (-0.02 to 0.00) |
|  | 12 months n=871 | 0.88 (0.16) | 0.88 (0.17) | 0.00 (-0.01 to 0.02) |

^$^ Adjusted for baseline alcohol consumption, AUDIT-C, age, sex, education, self-efficacy and EQ5D
