# Supplementary material for: On-line Randomized Controlled Trial of an Internet Based Psychologically Enhanced Intervention for People with Hazardous Alcohol Consumption
Source: PLoS One. 2011 Mar 9;6(3):e14740. doi: 10.1371/journal.pone.0014740 (PMC3052303; doi:10.1371/journal.pone.0014740)
Supplement: Table S5 — Secondary outcome measures (assessed in 1:4 participants). (0.05 MB DOC) [file pone.0014740.s009.doc]

|  |  | **Mean**  Intervention | Control | **Adjusted difference (intervention-control) of means (95%CI)^$^** |
| --- | --- | --- | --- | --- |
| **AUDIT** | Baseline n=1,991 | 18.8 (7) | 18.7 (7) | - |
|  | 1 month n=485 | 16.0 (8) | 16.2 (7) | 0.08 (-0.58 to 0.75) |
|  | 3 months n=857 | 15.2 (8) | 15.6 (7) | -0.06 (-0.58 to 0.50) |
|  | 12 months n=216 | 13.8 (6) | 15.2 (7) | -0.15 (-1.39 to 1.05) |
|  |  |  |  |  |
| **APQ** | Baseline n=1,990 | 6.6 (4) | 6.7 (4) | - |
|  | 1 month n=501 | 4.6 (4) | 4.8 (4) | 0.08 (-0.29 to 0.46) |
|  | 3 months n=879 | 4.0 (4) | 4.3 (4) | -0.25 (-0.58 to 0.10) |
|  | 12 months n=210 | 3.3 (3) | 2.8 (3) | 0.32 (-0.36 to 1.01) |
|  |  |  |  |  |
| **LDQ** | Baseline n=1,975 | 9.1 (6) | 8.7 (6) | - |
|  | 1 month n=529 | 6.6 (4) | 6.8 (5) | -0.10 (-0.69 to 0.46) |
|  | 3 months n=866 | 6.7 (5) | 6.1 (5) | **0.50 (0.01 to 0.95)** |
|  | 12 months n=220 | 6.0 (5) | 6.1 (5) | -0.14 (-1.12 to 0.66) |
|  |  |  |  |  |
| **CORE-OM** | Baseline n=926 | 1.3 (0.7) | 1.3 (0.7) | - |
| (Phase 1) | 1 month n=467 | 1.0 (0.7) | 0.9 (0.8) | 0.02 (-0.05 to 0.09) |
|  | 3 months n=364 | 1.0 (0.7) | 0.9 (0.6) | 0.08 (-0.01 to 0.16) |
|  | 12 months | - | - | - |
|  |  |  |  |  |
| **CORE-10** | Baseline n=1,050 | 16.3 (5) | 16.6 (5) | - |
| (Phases 2 & 3) | 1 month | - | - | - |
|  | 3 months n=453 | 14.5 (4) | 14.9 (4) | 0.00 (-0.66 to 0.63) |
|  | 12 months n=192 | 14.7 (4) | 14.5 (4) | 0.35 (-0.60 to 1.27) |

^$^ Adjusted for baseline alcohol consumption, AUDIT-C, age, sex, education, self-efficacy and EQ5D
