# Supplementary material for: On-line Randomized Controlled Trial of an Internet Based Psychologically Enhanced Intervention for People with Hazardous Alcohol Consumption
Source: PLoS One. 2011 Mar 9;6(3):e14740. doi: 10.1371/journal.pone.0014740 (PMC3052303; doi:10.1371/journal.pone.0014740)
Supplement: Table S6 — Subgroup analyses adjusting for baseline values. (0.07 MB DOC) [file pone.0014740.s010.doc]

| **Alcohol consumption in last week @ 1 month (*TOT-AL)*** | | | | | | | | |
| --- | --- | --- | --- | --- | --- | --- | --- | --- |
|  |  | | n | | | Adjusted ratio (intervention : control) of geometric means (95%CI)^$^ | P for interaction | |
| **Gender** | Males | | 844 | | | 0.99 (0.86 to 1.13) | 0.94 | |
|  | Females | | 1,223 | | | 0.98 (0.88 to 1.09) |
| **Baseline alcohol consumption (TOT-AL)** | Low/ Normal  (≤50♂ /35♀ units per wk) | | 880 | | | 1.01 (0.89 to 1.16) | 0.54 | |
|  | High  (>50♂ /35♀ units per wk) | | 1,187 | | | 0.96 (0.86 to 1.07) |
| **Education** | No degree | | 965 | | | 0.97 (0.85 to 1.10) | 0.74 | |
|  | Degree | | 1,102 | | | 0.99 (0.89 to 1.11) |
| **Self-efficacy** | Low (1,2,3) | | 1,493 | | | 0.98 (0.89 to1.08) | 0.96 | |
|  | High (4,5) | | 574 | | | 0.98 (0.84 to 1.15) |
| **Intentions** | Low (1,2,3) | | 773 | | | 0.98 (0.85 to 1.12) | 0.97 | |
|  | High (4,5) | | 1,294 | | | 0.98 (0.88 to 1.09) |
| **Alcohol consumption in last week @ 3 month (*TOT-AL)*** | | | | | | | | |
|  |  | | n | | Adjusted ratio (intervention : control) of geometric means (95%CI)^$^ | | | P for interaction |
| **Gender** | Males | | 1,376 | | 0.99 (0.89 to 1.10) | | | 0.30 |
|  | Females | | 2,153 | | 1.06 (0.97 to 1.16) | | |
| **Baseline alcohol consumption (TOT-AL)** | Low/ Normal  (≤50♂ /35♀ units per wk) | | 1,451 | | 1.10 (0.99 to 1.23) | | | 0.11 |
|  | High  (>50♂ /35♀ units per wk) | | 2,078 | | 0.99 (0.91 to 1.08) | | |
| **Education** | No degree | | 1,616 | | 0.97 (0.88 to 1.07) | | | 0.10 |
|  | Degree | | 1,913 | | 1.09 (0.99 to 1.19) | | |
| **Self-efficacy** | Low (1,2,3) | | 2,580 | | 1.04 (0.96 to 1.12) | | | 0.84 |
|  | High (4,5) | | 949 | | 1.02 (0.90 to 1.16) | | |
| **Intentions** | Low (1,2,3) | | 1,286 | | 1.05 (0.94 to 1.17) | | | 0.67 |
|  | High (4,5) | | 2,243 | | 1.02 (0.93 to 1.11) | | |
| **Alcohol consumption in last week @ 12 month (*TOT-AL)*** | | | | | | | | |
|  | |  | | n | Adjusted ratio (intervention : control) of geometric means (95%CI)^$^ | | | P for interaction |
| **Gender** | | Males | | 336 | 1.03 (0.81 to 1.30) | | | 0.60 |
|  | | Females | | 518 | 0.95 (0.78 to 1.15) | | |
| **Baseline alcohol consumption (TOT-AL)** | | Low/ Normal  (≤50♂ /35♀ units per wk) | | 362 | 1.01 (0.80 to 1.27) | | | 0.79 |
|  | | High  (>50♂ /35♀ units per wk) | | 492 | 0.97 (0.80 to 1.18) | | |
| **Education** | | No degree | | 352 | 1.03 (0.82 to 1.30) | | | 0.72 |
|  | | Degree | | 502 | 0.98 (0.80 to 1.18) | | |
| **Self-efficacy** | | Low (1,2,3) | | 626 | 0.98 (0.82 to 1.17) | | | 0.99 |
|  | | High (4,5) | | 228 | 0.98 (0.74 to 1.31) | | |
| **Intentions** | | Low (1,2,3) | | 288 | 1.04 (0.81 to 1.35) | | | 0.63 |
|  | | High (4,5) | | 566 | 0.97 (0.81 to 1.16) | | |

^$^ Adjusted for baseline alcohol consumption, AUDIT-C, age, sex, education, self-efficacy and EQ5D

Gender, TOT-AL and education were pre-specified variables for investigation of interactions; self-efficacy and intentions were included later
