# Supplementary material for: On-line Randomized Controlled Trial of an Internet Based Psychologically Enhanced Intervention for People with Hazardous Alcohol Consumption
Source: PLoS One. 2011 Mar 9;6(3):e14740. doi: 10.1371/journal.pone.0014740 (PMC3052303; doi:10.1371/journal.pone.0014740)
Supplement: Table S7 — Reported alcohol consumption in last week (units) by randomised group: alternative analyses allowing for missing outcome data. (0.04 MB DOC) [file pone.0014740.s011.doc]

| **Time point and method** | **Observations included in analysis** | **Geometric mean (SD)** | | **Adjusted ratio (intervention / control) of geometric means (95%CI)^$^** |
| --- | --- | --- | --- | --- |
|  |  | **Intervention** | **Control** |  |
| **Baseline** | 7,935 | 46.3 (31.8) | 45.7 (30.6) | - |
| **1 month** |  |  |  |  |
| Observed data | 2,067 | 27.1 (23.1) | 27.1 (22.5) | 0.98 (0.90 to 1.07) |
| Last observation carried forward | N/A^**^ |  |  |  |
| Multiple imputation* | 3,746 | 27.7 (23.6) | 28.8 (23.9) | 0.96 (0.88 to 1.05) |
| **3 months** |  |  |  |  |
| Observed data | 3,529 | 26.4 (23.0) | 25.6 (21.5) | 1.03 (0.97 to 1.10) |
| Last observation carried forward^†^ | 4,280 | 27.1 (23.6) | 24.5 (20.6) | 1.04 (0.98 to 1.11) |
| Multiple imputation* | 7,935 | 27.1 (23.6) | 26.0 (21.8) | 1.04 (0.96 to 1.12) |
| **12 months** |  |  |  |  |
| Observed data | 854 | 22.0 (20.0) | 23.5 (21.0) | 0.99 (0.85 to 1.15) |
| Last observation carried forward^†^ | 2,108 | 24.5 (22.3) | 24.5 (21.9) | 1.02 (0.93 to 1.11) |
| Multiple imputation* | 2,652 | 23.8 (21.6) | 23.6 (21.1) | 0.99 (0.82 to 1.18) |

* Imputation model includes baseline, 1, 3, and 12-month TOT-AL, AUDIT-C, age, sex, education, self-efficacy, EQ5D and website use variables

^$^ Adjusted for baseline alcohol consumption, AUDIT-C, age, sex, education, self-efficacy and EQ5D

^**^ Baseline not carried forward because of its large difference from follow-up values

^†^ Carried forward 751 1-month values for 3-month outcome (3,529+751=4,280) and 1,254 3m values for the 12m outcome (854+1,254=2,108)
