# Supplementary material for: On-line Randomized Controlled Trial of an Internet Based Psychologically Enhanced Intervention for People with Hazardous Alcohol Consumption
Source: PLoS One. 2011 Mar 9;6(3):e14740. doi: 10.1371/journal.pone.0014740 (PMC3052303; doi:10.1371/journal.pone.0014740)
Supplement: Table S8 — Effect of intervention on reported alcohol consumption in last week (units): sensitivity analyses allowing for missing data. (0.05 MB DOC) [file pone.0014740.s012.doc]

| **Sensitivity analysis** | **Non-responders differ in** | **Assumed difference between non-responders and responders** | **Adjusted ratio (intervention / control) of geometric means (95%CI)^$^** |
| --- | --- | --- | --- |
| ***1 month (n=2,067)*** | | | |
| **Moderate** | **Both arms equally** | 50% | 1.00 (0.92 to 1.09) |
|  | **Only control arm** | 25% | 0.84 (0.77 to 0.91) |
|  | **Only intervention arm** | 25% | 1.17 (1.07 to 1.27) |
| **Extreme** | **Both arms equally** | -50% | 0.95 (0.87 to 1.04) |
|  |  | 75% | 1.01 (0.93 to 1.10) |
|  | **Only control arm** | -50% | 1.61 (1.48 to 1.76) |
|  |  | 50% | 0.74 (0.67 to 0.80) |
|  | **Only intervention arm** | -50% | 0.58 (0.53 to 0.63) |
|  |  | 50% | 1.34 (1.23 to 1.46) |
| ***3 months (n=3,529)*** | | | |
| **Moderate** | **Both arms equally** | 50% | 1.07 (1.00 to 1.14) |
|  | **Only control arm** | 25% | 0.92 (0.86 to 0.98) |
|  | **Only intervention arm** | 25% | 1.18 (1.10 to 1.26) |
| **Extreme** | **Both arms equally** | -50% | 0.97 (0.90 to 1.04) |
|  |  | 75% | 1.08 (1.01 to 1.16) |
|  | **Only control arm** | -50% | 1.47 (1.37 to 1.57) |
|  |  | 50% | 0.84 (0.78 to 0.90) |
|  | **Only intervention arm** | -50% | 0.68 (0.63 to 0.73) |
|  |  | 50% | 1.31 (1.23 to 1.41) |
| ***12 months (n=854)*** | | | |
| **Moderate** | **Both arms equally** | 50% | 0.99 (0.86 to 1.15) |
|  | **Only control arm** | 25% | 0.81 (0.70 to 0.94) |
|  | **Only intervention arm** | 25% | 1.21 (1.04 to 1.40) |
| **Extreme** | **Both arms equally** | -50% | 0.98 (0.85 to 1.14) |
|  |  | 75% | 1.00 (0.86 to 1.15) |
|  | **Only control arm** | -50% | 1.83 (1.58 to 2.12) |
|  |  | 50% | 0.69 (0.60 to 0.80) |
|  | **Only intervention arm** | 50% | 1.42 (1.23 to 1.65) |
|  |  | -50% | 0.53 (0.46 to 0.62) |

^$^ Adjusted for baseline alcohol consumption, AUDIT-C, age, sex, education, self-efficacy and EQ5D
