# Supplementary material for: On-line Randomized Controlled Trial of an Internet Based Psychologically Enhanced Intervention for People with Hazardous Alcohol Consumption
Source: PLoS One. 2011 Mar 9;6(3):e14740. doi: 10.1371/journal.pone.0014740 (PMC3052303; doi:10.1371/journal.pone.0014740)
Supplement: Table S9 — Causal effects of using the intervention website on reported alcohol consumption in last week (units). (0.03 MB DOC) [file pone.0014740.s013.doc]

| Time point | Adjusted ratio (intervention / control) of geometric means (95%CI)^$^ | |
| --- | --- | --- |
|  | In compliers (complier average causal effect) | In those who downloaded 100 pages (instrumental variable methods ) |
| 1 month (n= 3,746)^ | 0.95 (0.85 to 1.07) | 0.94 (0.81 to 1.09) |
| 3 month (n=7,935)^ | 1.05 (0.95 to 1.16) | 1.06 (0.94 to 1.19) |
| 12 month (n=2,652)^ | 0.98 (0.77 to 1.14) | 0.97 (0.74 to 1.29) |

^$^ Adjusted for baseline alcohol consumption, AUDIT-C, age, sex, education, self-efficacy and EQ5D

^ n obtained from the multiple imputation model
