# Supplementary material for: On-line Randomized Controlled Trial of an Internet Based Psychologically Enhanced Intervention for People with Hazardous Alcohol Consumption
Source: PLoS One. 2011 Mar 9;6(3):e14740. doi: 10.1371/journal.pone.0014740 (PMC3052303; doi:10.1371/journal.pone.0014740)
Supplement: Table S10 — Summary of costs incurred in developing the intervention and comparator (2008 costs). (0.03 MB DOC) [file pone.0014740.s014.doc]

|  | **Intervention (£)** | **Control (£)** |
| --- | --- | --- |
| **Original development of DYD intervention** |  |  |
| Clinical and research academic time and on costs | 33,421 | n/a |
| Programming costs, publicity etc | 33,969 | n/a |
| **Development of intervention for trial** |  |  |
| Clinical and research academic time and on cost | 6,741 | 304 |
| User panel | 640 | 320 |
| Programming costs etc | 27,425 | 1,645 |
| Web hosting and maintenance costs | 1,121 | 1,121 |
| **TOTAL** | **107,317** | **3,390** |
